# Supplementary figures and images for: Spontaneous single-nucleotide substitutions and microsatellite mutations have distinct distributions of fitness effects
Source: PLoS Biol. 2024 Jul 1;22(7):e3002698. doi: 10.1371/journal.pbio.3002698 (PMC11244821; doi:10.1371/journal.pbio.3002698)

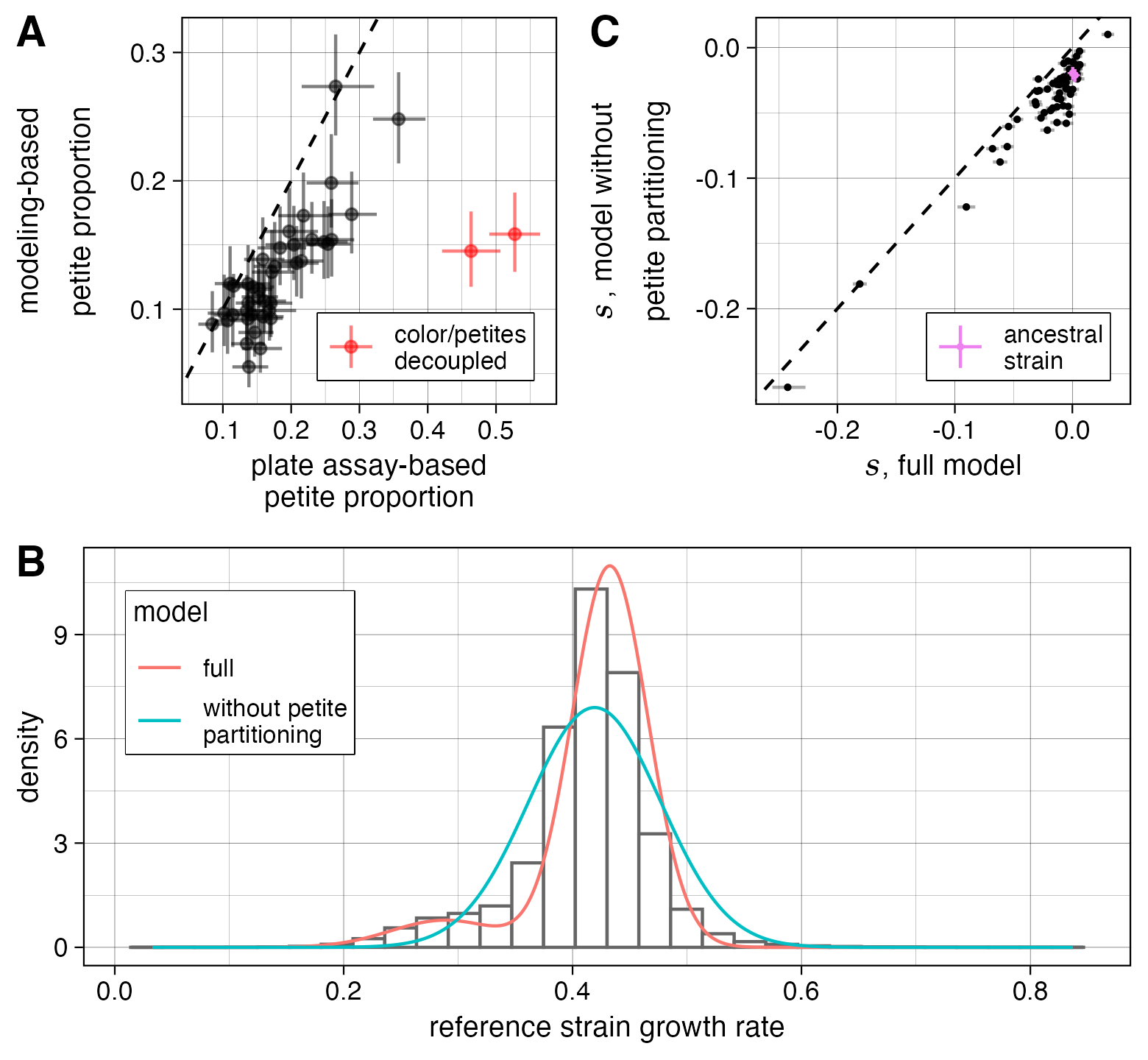

Supplement: S1 Fig — (A) Petite proportion estimated for strains from [38] using plate-based colony color assay versus by modeling observed colony growth rates in the microscope assay as a mixture of Gaussians. Error lines represent 95% confidence intervals; dashed line shows a 1:1 correspondence. Each point represents estimates for a single strain on a single experimental day (with error bars based on replicates across microscope plate wells). Two data points in red are for a strain in which colony color and colony size were decoupled. (B) A histogram of growth rates of the ancestral reference strain in all wells in which it was co-cultured with 2,000-generation MAH strains. Red line shows the distribution estimated by the best-fit model of the distribution of growth rates for this strain in the full model (including a distribution of petites); blue line shows distribution estimated by the model in which the distribution of growth rates is not partitioned into petite and non-petite growth rates. Note that although distributions are shown overlaid on raw reference strain growth rate measurements, the models that produced the distribution parameters were based on differences between reference strain and MA strain growth rates (see Methods). (C) Mutational effects for strains from Fig 1 estimated either by the full model of MA strain s effects described in the text, or by a model that does not include a petite population in any of the strains. Error lines represent 95% confidence intervals; dashed line shows a 1:1 correspondence. Two ancestral control strains included in the experiments (purple points) have mutational effects whose confidence intervals overlap with 0 when petites are accounted for, but not when they are ignored. (TIFF) [file pbio.3002698.s011.tiff]

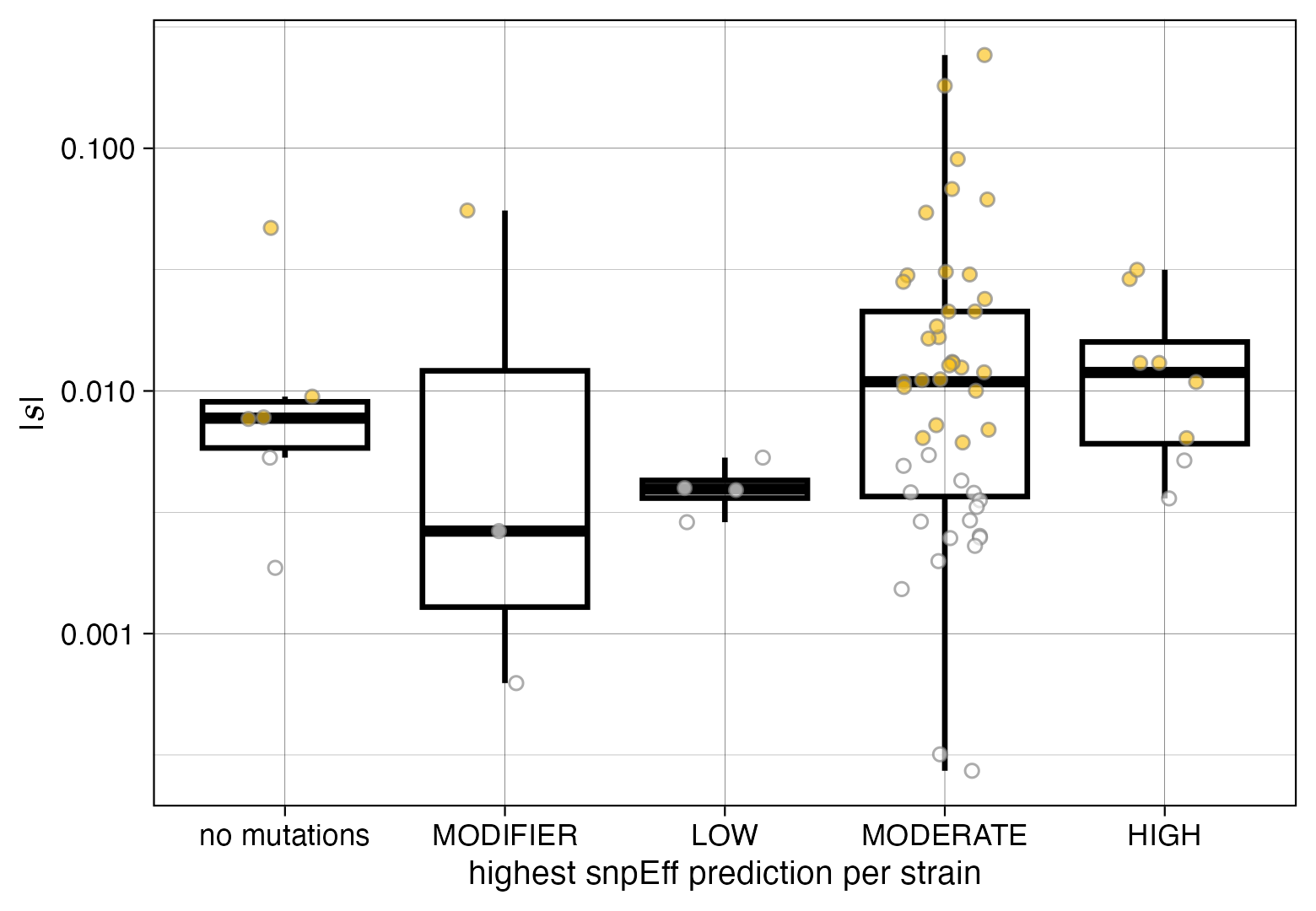

Supplement: S2 Fig — The absolute value of the MLE of the selection coefficient of each 2,000-generation MA strain, with strains grouped by the effect of the highest putative effect non-repeat mutation, as predicted by snpEff, found in each one. Points are colored yellow if their s value differs significantly from the ancestor at an FDR of 0.05. (TIFF) [file pbio.3002698.s012.tiff]

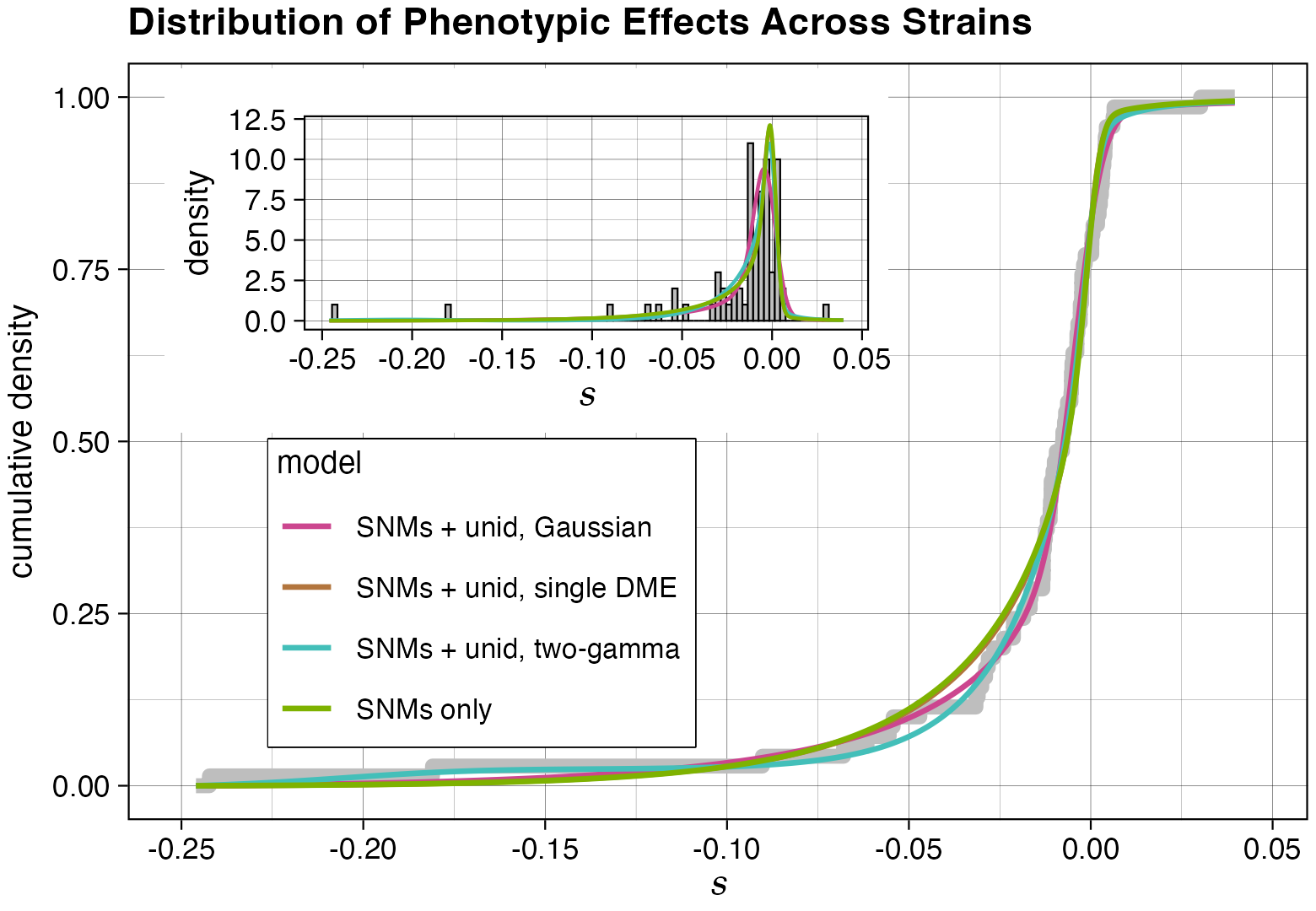

Supplement: S3 Fig — The cumulative density function of the fit of each DME model to all individual MA strain mutational effects s. Inset: histogram of mutational effects with probability density functions of the models overlaid. To account for the effect of experimental noise on the estimates of s, the model density function is shown convolved with a Gaussian noise kernel with a variance that is the mean of the error variances of each strain’s mutational effect estimate. SNMs + unid, Gaussian model as in Fig 2. (TIFF) [file pbio.3002698.s013.tiff]

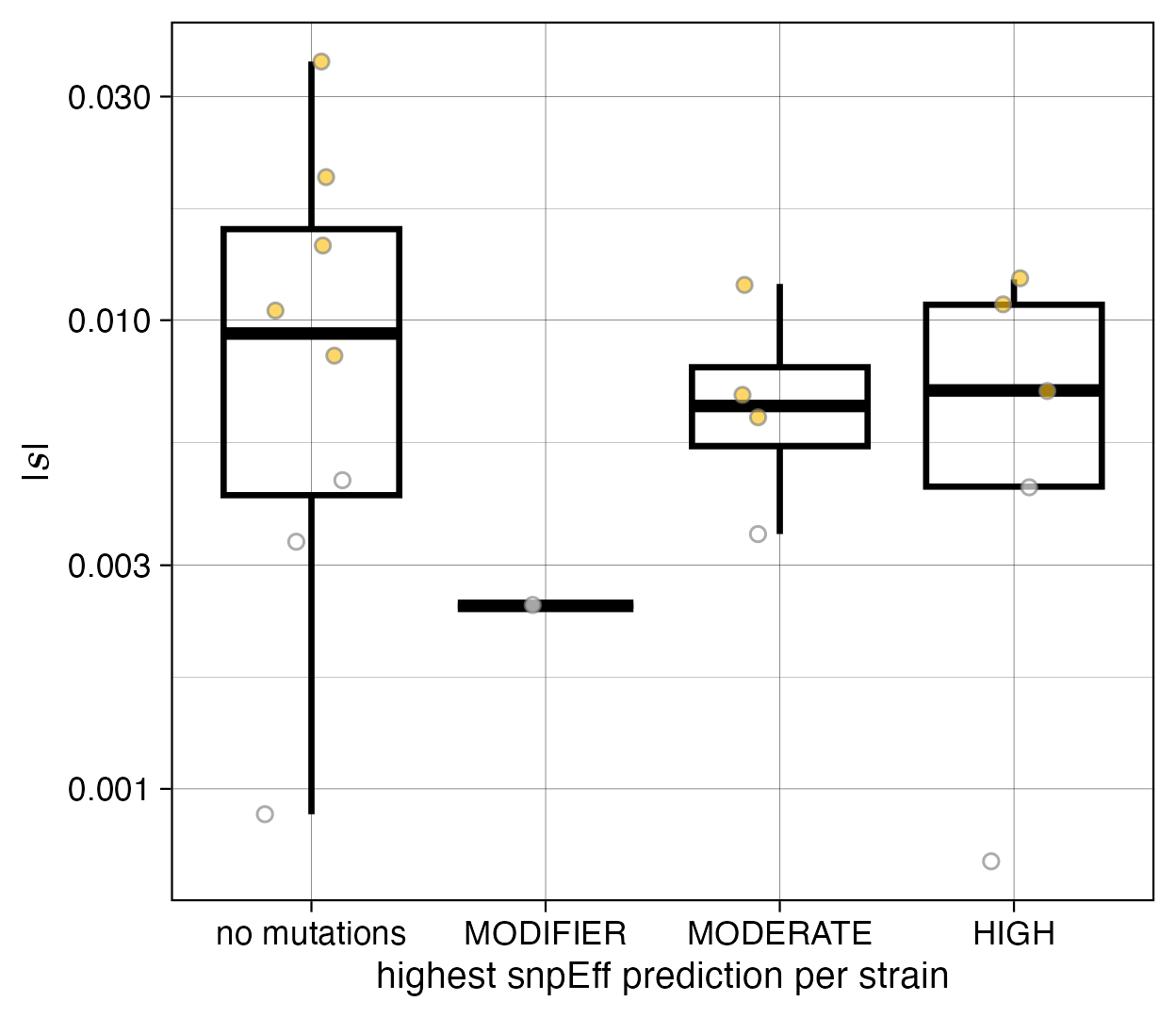

Supplement: S4 Fig — The MLE of the absolute value of the selection coefficient of each msh3Δ MA strain, with strains grouped by the effect of the highest putative effect SSR mutation, as predicted by snpEff, found in each one. Points are colored yellow if their s value differs significantly from the ancestor at an FDR of 0.05. (TIFF) [file pbio.3002698.s014.tiff]
