# Supplementary material for: Spontaneous single-nucleotide substitutions and microsatellite mutations have distinct distributions of fitness effects
Source: PLoS Biol. 2024 Jul 1;22(7):e3002698. doi: 10.1371/journal.pbio.3002698 (PMC11244821; doi:10.1371/journal.pbio.3002698)
Supplement: Attachment — Submitted filename: Reviewer Response V1.pdf [file pbio.3002698.s015.pdf]

Point-by-point response to reviews (review text in black, responses in blue)

Reviewer 1:

Plavskin et al. attempt to estimate the distribution of fitness effects of de novo mutations in Saccharomyces cerevisiae. This question is indeed important for understanding the dynamics of evolution, quantitative traits' architecture, genetic load of extant populations, cancer development, etc. The authors generally correctly explain shortcomings of former studies. I have not noticed in the text any generalization about the distribution(s) of mutational effects reported until now with yeast so that the authors could conclude to what extent their finds confirm or contradict those already known. I believe the main finding of the study (s mostly negative and small) is correct, I would be highly surprised if something much different was found. It does not mean that the present experiment simply repeats those done earlier. The strongest aspect of the study is the application of highly repeated measurements of microcolony growth rate which helps to get precise estimates. The design of the experiment, including the use of controls, appears correct. Computational analyses built on earlier ones but try to introduce new constraints and are carefully done. Still, there are several non-trivial issues that need to be explained before the paper is published.

We thank the reviewer for a close reading of this manuscript, and address these issues below.

1) "Variation in petite numbers across samples can arise from chance events that cause different numbers…" A single loss of the mitochondrial function is probably "random". But, some strains have repeatedly about 10% while others up to 30%, even if two different methods are applied, colony color and distribution fitting. Kind of stabilized randomness. The authors say:" these strains differed from each other by only ~2 mutations on average, large variation in the proportion of petites across these strains was not likely to be explained by genetic differences among the strains." First of all, mutations were few but different. If not the few known mutations then what caused this variation. Epigenetics? Hardly. Repeatable elements, plasmids, viruses? Don't know. My guess would be mutations in mtDNA of the highly inbred lines. Was mtDNA analyzed with comparable accuracy to the nuclear DNA? Is it just my curiosity? The authors should suggest some explanation, at stake is the question how representative are their strains for "normal" yeast.

Most MA strains used in our work had similar proportions of petites, around 15%, which is consistent with estimates for other lab strains of yeast, e.g. W303 and SK (Dmitrov *et al*., 2009) and FY4 (Li *et al*., 2018). Nonetheless, it is indeed the case that some strains had repeatably higher or lower proportions of petites than the ancestral strains (6 strains >20%, 1 strain <10%). We agree that if this is indeed caused by a genetic factor, it would be a very interesting subject for further investigation, although this question lies outside the confines of the current study, which focuses on the overall distribution of mutational effects. The main goal of modeling petite proportions in our work was to prevent stochastic variation in founding culture petite proportions from influencing our measurements of the selection coefficient.

2) The petite proportions are extremely high. Why apparently none strain was put onto non-fermentable medium to confirm these were indeed petites? Petites are really slow growers so they are quickly overgrown by respiring cells in normal strains (about 1% of petites). With 0.1-0.3 proportion, the petite mutation rate must have been up to 0.5. If so, there were no two types of colonies; "petites" versus "non-petites". Rather there was a rising fraction of pure "petites" (arriving after last respiring within colony mutated) against a declining fraction of petite-respiring mixed colonies of evolving composition (with selection favoring non-petites but mutation killing them). Can you model all this mess to extract TRUE non-petite s? Or perhaps admit that petites are part of every strain and calculate overall s for all the beasts forming it? (As the poor "batchers" do.)

We agree that the petite-generating process is complex, and we are not attempting to model this complexity in our study. When considering how fast petite cells are overtaken by non-petite cells, it is important to remember that our cultures are not experiencing constant exponential growth before the growth-rate measurement; significant amounts of time spent in stationary phase (likely the last ~12-24 hours before the experiment) will change the tradeoffs experienced by petite cells. Previous studies in laboratory yeast strains have found *de novo* petite generation rates to be on the order of 5% per generation in a laboratory strain with an overall petite frequency of ~45% (Dmitrov *et al.* 2009). Moreover, in this study we measure the growth rate of petites to be ~2/3 that of non-petites, so the per-generation petite-generation rate of significantly lower than 5% can maintain a 15% petite proportion.

Most importantly, however, in our work, the petite generation process primarily matters in the sense of producing some number of petite microcolony founder cells that we then exclude from further analysis. Petite generation during the growth of the microcolonies themselves does not have large effects on the growth rate of the overall colony, since on the scale of a single colony grown for many generations, the mean growth rate is heavily weighted by the much larger number of fast-growing cells.

As we discuss in the paper, one of our chief concerns is that the variation in petite number, at least among some strains, may not represent genetic variables, but rather be the result of jackpotting events during culture. We indeed have evidence of this occurring, as well as evidence that it would result in incorrect estimates of growth rate differences between strains: differences in petite proportions between the reference strain and two ancestral control strains cause non-zero *s* values to be estimated in these strains when the petite proportion is not modeled.

3) "(Pearson correlation coefficient = 0.83) indicating that microcolony growth rate data can be used directly to partition growth rates of petite and respiring colonies." Squared becomes not that impressive. Errors introduced here may account for much of the variation in the s of respiring cells. So, think of the advice from the previous point.

We agree that there is far from a perfect correlation between the microscopy-based and plate-based measurements of petite rate. However, an important point is that the (relatively) low

numbers of colonies counted in these assays (~200 per plate, so average petite numbers are only around ~30 colonies, replicated 2x) mean we expect relatively high error in the estimated petite proportion for each strain. In addition, most strains have similar petite proportions. As a result, high correlation with the plate-based assays would not be expected even if there were no error at all in the microscopy-based assay (although of course that error does exist, and is reported in S1 Table)

4) "diploid parent strains that accumulated mutations over the course of 2000 generations accumulating an average of ~8 SNMs." First, were these mutations in coding regions, non-synonymous and with likely non-negligible protein effect? If not, why do the authors use these numbers in their models? Yes, there was a famous paper in Nature claiming that synonymous may have handsome s, but I prefer the old framework. Second, among the parental diploids the scores of mutations must have differed (4-12?), was there any correlation between these counts and average s of the two derived haploids?

We thank the reviewer for suggesting a deeper analysis of SNM locations and effects. We have now sequenced the haploid strains whose phenotypes we have measured, and present the results in the revised manuscript. Most importantly, we find that several strains with either 0 identified mutations or only synonymous/intergenic mutations have *s* values that deviate significantly from 0. This finding strengthens our conclusion that additional, non-SNM mutations are impacting growth rate in those strains.

5) "Consistent with our previous finding, the 'Gaussian' model, which modeled SNMs and unidentified mutations as having two independent DMEs, provided the best fit to the data". Can you think of an analysis of empirical data in which adding two more parameters to a model would not improve the fit?

The reviewer is of course correct on this point, but it is important to note that this is why it is critical to analyze whether the improved fit to the data is statistically significant when accounting for the extra parameters. The large negative change in AIC in the model with the extra parameters allows us to conclude that the improvement is not merely due to the addition of parameters. However, more broadly, we agree that there are important caveats to interpreting the modeling data on its own. This is why we believe that the data in our paper directly showing growth-rate differences in strains containing SSR mutations but no non-repeat SNMs are critical for showing that SSRs very likely affect growth rate.

6) "These strains accumulated mutations over the course of 200 generations, and contain an average of 1.8 SSR mutations each, the majority of which are deletions of a single repeat unit." Again, where they were located, what effect could they have. In the case of SSR the question is even more important because here "17% of strains have a significant increase in growth rate, and 44% have a significant decrease in growth rate". How can I accept it without seeing the mutations. MSH3 is known to stabilize many repetitive sequences, especially telomeres, those regular and their remnants scattered over chromosomes. NGS tells nothing of mutations within them and about many types of possible chromosome-segment mutations in mismatch repair

deficient strains. I do not believe in the whole story about SSR, including the title. Yes, I can not prove I am right and you are wrong.

We have updated the manuscript to report the putative impacts of the SSR mutations in our strains. Importantly, to do this we sequenced our 2000-generation MA strains and identified a subset of the SSR mutations in those strains. As we now report, among these SSR mutations, about half are intergenic mutations, ~⅓ are 'moderate'-effect mutations (e.g. missense variants and in-frame deletions and insertions), and ~⅙ are 'high'-effect mutations (e.g. frameshift mutations). Notably, among MA strains with *s* values significantly deviating from 0 but with no identified SNMs, we identified moderate- or high-effect SSR mutations in most strains.

We agree that we cannot exclude the possibility of mutations in telomere, centromere, or LTR sequences having effects on yeast growth that contribute to non-0 selection coefficients in strains lacking SNMs, and have revised the Discussion (lines 629-632) to make this point more clear in our paper. However, we do not believe that such mutations would likely explain most missing variation in *s* among strains for the following reasons:

1. Although telomeres (and perhaps centromeres) are also stabilized by MSH3, these sequences make up a smaller proportion of the genome overall: together, telomeres and centromeres make up ~1% of the yeast genome, whereas SSR sequences make up ~10%.
2. In the yeast genome, SSRs are usually found in close proximity to protein-coding genes or (primarily in the case of 3-bp repeats) inside protein-coding genes.
3. LTRs make up ~3.5% of the yeast genome, but we expect the rate of SNMs and small indels in these sequences to be similar to that of other parts of the genome.

7) "potential aneuploidies were excluded from our analysis", from diploids, but meiosis could introduce them again especially in these pretty awkward strains

We thank the reviewer for pointing out this issue. Indeed, sequencing revealed that 2 of the 70 2,000-generation MA-derived haploid strains used in our analysis are aneuploid. We do not expect these strains to have skewed the data in such a way as to modify the key findings of our study, especially since they are not among the SNM-free strains at the core of the argument about the effects of SSR mutations. However, if the reviewer and editors deem it advisable, we would be happy to rerun the full DFE modeling without including these strains.

8) "Two a mating type spores from a full tetrad derived from the diploid ancestor". It would be helpful to know about the frequency of full uniforms tetrads or 2:2 segregations with lethals or slow growers. There was a study based on that. It lacked fancy models but provided results parallel to those reported here,
https://urldefense.proofpoint.com/v2/url?u=https-3A__doi.org_10.1093_genetics_159.2.441&d=DwIGaQ&c=slrrB7dE8n7gBJbeO0g-IQ&r=18XU5COieVxJdStm6YvlTcCmIcGz4K6MigTjpypY_mE&m=Qn0z8Rn0I7daCFmGYJgrHA2Y9GN9aipt-VewQvzd6aSfWqCHsMcB2ZbPoWi45-Z7&s=t708kf2PNkcX8QMW8xCkdbN38LT-yRSPSdwfrpYygK4&e= .

We thank the reviewer for pointing out this paper; these results are indeed parallel to ours, and we have cited it in our discussion.

We do not have data for growth rates of tetrads derived from diploid MA lines, with the exception of the ancestor. However, Hall and Joseph (2010) reported that ~10% of 2000-generation diploid MA lines contained mutations that were lethal in haploids. Of course, these lethal mutations are by necessity excluded from our study.

We believe performing analysis of segregation of the growth-rate phenotypes in tetrads derived from diploid MA parents using our high-sensitivity assay may allow us to estimate the relative frequencies of neutral vs. strong-effect mutations in our strains with higher precision, and would be an interesting direction for future work.

Reviewer 2:

The authors address challenges in the field of evolutionary genetics and fitness by using a sensitive assay and modeling to determine the effects of mutations on growth in S. cerevisiae. Their rationale and findings are clearly communicated, and address their 3 stated goals of determining the frequency of deleterious mutations, what proportion of the genome that affects growth when mutated, and how different classes of mutations affect growth. Their findings that many mutations are deleterious, but with very small effect sizes, make an important contribution to reconcile prior studies that differed in their findings because many of these small effect sizes are not captured in other fitness assays. Their method and models are a powerful tool for investigation of growth phenotypes, particularly by addressing the contribution of petites. This study provides resources useful to the yeast genetics field and results with broad impact across the field of evolutionary biology.

My only minor comments, which do not necessitate revision but should be addressed if others deem revision or edits are needed, are to clarify figures labels. The labeling of Fig. 2B, in the panel and the written legend, was unclear - the label "without petites" seems to indicate that petites were excluded from the data used to generate the model, which is not the case. "Without partitioning," or "Single gaussian" and "Two gaussian" would make it clearer which line goes with which model as described in the main text. In general, adding legends showing color and label into the panels themselves would make reading and interpretation easier than searching for them in the written figure legends (e.g., how Fig 2B, S1 Fig B, and S2 Fig have the color legends within the panel).

We thank the reviewer and have made the suggested changes

Reviewer #3:
[identifies himself as David Houle]

This manuscript addresses the very interesting issue of the distribution of effects of spontaneous mutations on fitness. The strengths of this paper are many.

It leverages the many tools available in the yeast system in service of the ultimate goal. This enables the authors to preserve and label control strains, to account for the annoying tendency of yeast strains to throw off petite genotypes, and to obtain wonderful precision in the estimates of growth rates of individual clones. Finally, by disabling the slippage repair mechanism, they accumulated mutations in repeat numbers at rates large enough to provide a useful sample.

The authors are able to count single nucleotide mutations, and use those totals to put precise limits on their estimates of the DFE.

The results provide very useful estimates of the proportion of SNMs with positive and negative effects. The result that the most likely proportion of neutral mutation is very low is quite striking. The broad confidence limits on this estimate are disappointing, but the result itself is quite provocative, and will surely lead to additional work.

Some of the most useful results are the authors estimates suggesting the SSRs have substantial fitness effects averaging 0.3% per variant.   This is the first such estimate that I know of, and, as the authors lay out in the Discussion of practical importance.

There is one element missing from the analysis that I would really like to see if the authors can supply. There is no explicit test, that I can find, of whether the DFEs for SNMs and SSRs is different. The manuscript would be improved by attention to this problem.

We thank the reviewer for the positive comments about our manuscript.

One of the difficulties in *directly* testing whether the DFE of SNMs differs is the difficulty of estimating precise SSR mutation rates. This analysis could perhaps be done (with a highly imperfect estimate of the mutation rate) by estimating the DFE in SNM-free *msh3*Δ mutation accumulation strains. However, the average number of mutations in these strains is low, as are the typical mutational effects measured. This leads to large uncertainties in estimates of the DFE for SSRs in these strains, and we have elected not to include this analysis in our paper.

However, importantly, we can *indirectly* test whether SNMs and SSRs appear to have different distributions of mutational effects by comparing the model that includes a distinct SSR effect distribution (the ‘Gaussian’ model in our paper) to one in which we allow for additional mutations, but constrain them to have the same DFE as that of SNMs. Our analysis (*S2 table*) shows that the ‘Gaussian’ model provides a significantly improved fit to the data after accounting for the additional parameters (ΔAIC = -3.17), whereas the model that allows additional mutations to have effects drawn from the same DFE as SNMs (‘single DFE’) only provides a moderate improvement to the fit to the data over the SNM-only model (ΔAIC = -0.89). This suggests that modeling the data using a separate distribution of effects for the ‘unidentified’

mutations improves model fit relative to a model that fits a second distribution of mutations drawn from the same DFE as the first. We have updated the text describing these results (lines 442-444) to make this point more clear.
